# On the Treatment of Tachycardia

**Published:** 1909-04

**Authors:** A. Goldscheider

**Affiliations:** Director of the Virchow Hospital at Berlin


### On the Treatment of Tachycardia

By Privy Councillor Professor A. GOLDSCHEIDER, M. D.
Director of the Virchow Hospital at Berlin

(*Folia Therapeutica*, October, 1908.)

TO obtain successful treatment of accelerated action of the heart it is necessary to clearly establish the cause of the attack. The physiological factors tending to the production of such a result must come under primary consideration during the formalities of a medical examination, otherwise a false idea of the condition of the heart may be arrived at.

These factors are severe physical exertion, nervous and mental excitement, and the taking of coffee or alcohol. With a healthy heart the increased frequency of the pulse after physical exertion soon quietens down, but where the heart is weak or where there has been unusually great or unaccustomed exertion, it may continue for a long time, during which it may chance to come under medical examination.

Palpitation induced by the mental excitement of a medical examination is of great importance. The causes of accelerated heart action may lie in the nervous mechanism of the heart as well as in the heart muscle itself. Regarding the latter, on the one hand its weakness, and on the other its increased irritability, may give rise to a heightened pulse-rate, so that, in cardiac muscular debility we commonly find during rest an increased pulse frequency which, by slight changes in position or continued movement, is still more increased.

Inflammatory conditions, such an endo-, myo- and pericarditis, likewise cause increased pulse frequency, and the influence of the nerves on the activity of the heart muscle is so intimate and far-reaching that in those cases of tachycardia which are provoked by either a condition of debility or of irritability of the heart muscle, the participation of the cardiac nerves manifestly cannot be excluded. It is expedient, however, on diagnostic and practical grounds, to differentiate between those cases which have their origin in the nervous system and such as are dependent on an affection of the heart itself. It is doubtful to which group such clinical forms as paroxysmal tachycardia should be referred, but we shall provisionally include in the nervous group those cases in which disease of the heart itself is not discernible, and which show during their course that this is probably the case. In a third group those cases will be included in which it is certain that the nervous mechanism, as well as the heart itself, is simultaneously affected. In cases of *tachycardia of nervous origin* those of a *functional* nature predominate, though they may be caused by organic changes in the nerves as well as by pathological changes in the brain, medulla, or vagus nerve. One calls to mind the quickened pulse of meningitis, of brain compression, acute bulbar paralysis and tabes dorsalis, also of compression of the vagus (by tuberculous glands) and vagal neuritis.

These *functional* nervous attacks occur in the following forms: psychogène emotionelle (hysterical), palpitation, neurasthenic, reflex, toxic, anemic, and finally as a pure cardiac neurosis, which is always combined with a vasomotor neurosis. An increased pulse frequency of nervous origin may be assumed to exist in the absence of symptoms of organic heart disease, more particularly when there are indications of neurasthenia, hysteria, or some alteration in the condition of the mind, or when some source of irritation is shown to exist, and the course of the tachycardia and the manner of its appearance demonstrate the nervous character of the disorder, as, for instance, the cessation of functional tachycardia during sleep as was observed by my assistant, Dr. Kroner.

Toxic tachycardia (and arrhythmia) and that occurring after infectious disease may belong to either the nervous or to the myogenic subdivision of the mixed myogenic forms.

I will discuss the separate forms of *myogenic or muscular* tachycardia later, but here would lay stress on the fact that the increased pulse frequency, which is undoubtedly caused by organic heart disease, may at any time be complicated by nervous and psychical influences. Increased heart action is not necessarily accompanied by the sensation of palpitation. In many cases the patients feel nothing at all, in others they complain of fluttering and a feeling of uneasiness in the cardiac region, and in a proportion of the cases the palpitation is felt simply as an inconvenient throbbing, or is accompanied by painful sensations or mental distress. This increased action may be combined with irregularity (arrhythmia).

Groups of cases occur in which quick or slow heart action takes place, and the physiological respiratory arrhythmia is abnormally increased, and it is mostly these to which functional nervous arrhythmia belongs. Intermission and extra-systolic action are rare and occur in isolated cases only. Not infrequently an unequal time interval occurs between the separate heart beats and in the arrhythmia incident to disease of the heart muscle the frequency of extra-systolic action is characteristic.

Certain states of the heart depending on myocarditis, atheroma of the aorta, syphilis, or even granular kidney are very often held to be of nervous origin, because the increased pulse-rate, the feeling of anxiety, pain and lack of objective heart symptoms lead to that supposition. A careful examination of the heart, however, including examination by the *x*-rays, would often guard against such an error. A positive finding of the condition of the heart would not, on the other hand, exclude the possibility of an accelerated heart being of a nervous character. All nervous, physical and reflex (*e. g.*, gastrointestinal and genital) agencies may be effective in either a sound or unsound condition of the heart muscle. Marked palpitation during active exercise is mostly a symptom of cardiac weakness. Many heart patients have quite an ordinary pulse so long as they remain quiet, but on slight locomotion or by merely assuming the erect attitude they get increased pulse beat and finally cardiac arrhythmia. But a not inconsiderable action of the heart may take place in nervous conditions also during exercise, mostly when such are combined with a certain sense of fatigue or some psychical excitement on the part of the patient. The treatment of nervous and that of myogenic tachycardia varies widely.

In the nervous forms real heart tonics, such as digitalis and strophanthus, are usually of little good; yet, unfortunately, preparations of digitalis are frequently prescribed to slow the pulse in tachycardia, the only result being the acquisition of dyspepsia.

The following measures are recommended for adoption as accessory to the treatment of the real underlying cause of tachycardia:

(1) *Rest in the Recumbent Position.*—It should be here noted that some neurasthenics cannot endure protracted rest, since they suffer nervous attacks, and on this account must be allowed occasional exercise.

(2) *Application of Cold to the Heart.*—This may be carried out by means of an icebag, or bottle filled with cold water, or by the use of Gaertner's coil, the latter being particularly recommended as it adapts itself so well to the chest wall, exercises no inconvenient pressure and by it the cold may be uniformly applied. The application of cold to the cervical region (nape of the neck) may be tried.

(3) *Mental composure* is of great importance not only in nervous but in every form of palpitation, as a close relation exists between mental conditions and the action of the heart; and it is in these conditions that sedatives are useful.

(4) *Sedatives.*—Preparations of the bromides, such as sodium bromide or a mixture of sodium, potassium and ammonium bromide, or the well-known effervescing bromide salt, or bromide tablets. Among the newer bromide preparations which may be recommended are: bromalin 3 to 5 grams a day, in powders of 1 gram each; bromipin, in doses of one teaspoonful three or four times a day, or from 30 to 50 grams as a clyster. Veronal in doses of 0.1 gram two or three times daily, sometimes exerts a soothing influence on general excitement, especially on that of the heart. Bromural (monobromisovalerianylurea) has also been much recommended in doses of 5 to 10 grains as a sedative drug, combining the effects of the bromides and valerians with the urea basis, which induces a healthy, dreamless sleep, from which the patient awakes fresh and invigorated. Huchard recommends quinine hydrobromide. Valerian preparations sometimes give excellent results, of which bornyval is worthy of special mention, as well as valerian tea or the tincture. Hydrocyanic acid should also be tried, the simplest form of which is the aqua amygdalarum amararum or as cherry laurel water (30 to 40 drops a day). The local application of menthol in the form of a stick or as an ointment, and inhalations of menthol dissolved in hot water are likewise useful in many cases.

(5) Should the accelerated nervous pulse be weak as well, we may draw on the preparations of caffeine (caffeine, caffeinæ-

sodio-benzoicum, caffeinæ-sodio-salicylas) as well as tincture of strophanthus. The extractum cacti grandiflori fluidum may be tried, 10 to 20 or 30 drops three times a day.

In violent action of the heart, which frightens the patient and causes great alarm, small doses of morphia, codeine or dionin may be necessary. At times the application of a pressure bandage (or Abbé's heart support) acts beneficially.

(6) *Gentle massage of the cardiac region,* abdomen and back, and the employment of electricity in its various forms (galvanism, alternating current baths and the high frequency current) are within the sphere of treatment to be adopted in all cases of nervous tachycardia.

(7) Lukewarm baths, 26° R. to 27° R (92° F. to 94° F.), with or without the addition of aromatic vegetable extracts.

As already remarked, the primary disease (neurasthenia, anemia, the uric acid diathesis) must be treated.

Where stomach, intestinal or sexual reflexes are the cause of tachycardia, they should be treated therapeutically. Fermentative changes or excessive gastric acidity should be treated with alkalies, or the stomach should be washed out and the diet carefully watched. An article which has often proved of the greatest service to me in conditions such as these and in reflex cardiac disturbance is camphora trita in doses of 0.1 gram three or four times daily, and camphora brom. 0.1 to 0.2 gram several times a day.

The occurrence of tapeworms in the intestinal canal may produce reflex tachycardia, and the taking of rich and heavy suppers may cause nocturnal palpitation. Moreover, the connection of gastric and intestinal affection with disturbance of cardiac innervation is not always merely reflex, the toxic effects of intestinal fermentation with pushing up of the diaphragm and consecutive mechanical influence on the heart being of greater importance.

(8) The intimate relation between tachycardia and sexual conditions is recognised, and among those coming under chief consideration are the period of puberty, especially in the female sex, the climacteric, dysmenorrhea, masturbation and diseases of the organs of generation (prostatitis, gynecological affections).

In treatment, the ailment which is the primary cause of the condition should always receive the first consideration. Amongst cases of nervous tachycardia, those accompanied by arterial constriction take an important place, the latter being caused by various conditions, as in the spastic angioneurosis of anemia, chlorosis and neurasthenia; on the other hand, toxic forms, such as the uric acid diathesis and the climacteric cardiac neurosis, are often combined with vascular spasm (Huchard). Besides the symptoms of a cardiac neurosis there are added pallor and coldness of the extremities, paresthesia, vertigo, polyuria, and tensely contracted arteries.

It is possible, though it has not been conclusively proved, that the acceleration of the pulse is the result of the contraction of the arteries, but it is more probable that the irritability of the heart and of the vessels has a common cause. At any rate, treatment directed to a lowering of the vasomotor tone is indicated, such as washing and sponging of the extremities, alternate hot and cold friction, dry rubbing and the exhibition of nitroglycerin, trinitrin and amyl nitrite. Similar treatment is also recommended for the real angina pectoris vasomotoria.

In paroxysmal tachycardia not all treatment is useless. It is said that pressure on the vagus in the neck may mitigate the attack. I have often tried it, and have obtained no measure of success, but I have seen a draught of cognac put an end to the attack, and in another case the artificial induction of retching movements brought the attack to a rapid termination. But in most cases the means usually adopted are without effect, and then the attack ceases suddenly without any apparent cause. Besides treating the stomach, the introduction of cocaine or, better still, novocaine (in 5 per cent. solution) with suprarenin into the nose should be tried. Huchard recommends sulphate of quinine and adrenalin in lowered arterial pressure, also the application of ethyl chloride to the neck and back, and hydrotherapy in the form of cold sponging of the whole surface of the body, and more particularly the vertebral column, followed by a hot pack to induce sweating. Deep inspirations, too, may be tried, which not infrequently give at least temporary relief. Mustard plasters to the epigastrium and back of the neck are well worthy of trial. In Basedow's disease digitalis is a very popular remedy.

I have never seen a case in which this has done good, on the contrary, the cardiac systole without being prolonged is increased in force, which adds still more to the patient's discomfort. It is only when muscular insufficiency and dilatation supervene on the ordinary symptoms of Grave's disease that anything can be expected of digitalis.

Among muscular anomalies we will consider in general those cases of tachycardia and arrhythmia which occur when there is evident pathological change in the heart, or which are combined with distinct signs of muscular insufficiency, or which, according to their kind (rapid extra-systole, or well-marked inequality of the pulse), permit us to recognise the myogenic type. As already noted, the first-named point is not decided, for nervous tachycardia and arrhythmia may occur in organic heart disease. Myogenic tachycardia (and arrhythmia) is in part a symptom of debility. We find it in cardiac muscular weakness, in myocarditis, arteriosclerosis, valvular heart disease, especially in mitral insufficiency, in dilatation of the heart, endo- and pericarditis and in contracted kidney. Toxic influences are of some importance, as well as the anatomical changes in the heart muscle. In cases of muscular arrhythmia the counting of the pulse should not be relied upon, but the rate of the cardiac contractions should be estimated by a careful auscultation of the heart itself, as owing to its lack of force a greater or less number of beats are not felt at the pulse. These missing pulse beats may amount to a half of the beats of the heart. It is here that heart tonics find their true sphere of action, and foremost among these is digitalis. It is common knowledge that there has been great difficulty in obtaining a constant preparation of the active principle of this drug, which varies according to the season when, and the source from which, it has been obtained and the manner in which, and the length of time, it has been kept.

The following preparations are worthy of recommendation: Digitalisatum, Bürger; dialysatum fol. digitalis, Golaz; crystalline digitoxin, Merck; and digitoxinum solubile, Cloetta; or digalen, which may be obtained commercially as an aqueous solution to which glycerine has been added.

Generally speaking digalen causes less dyspepsia than the other preparations of digitalis, and has the further advantage that it may be injected subcutaneously, into the muscles or even into the venous blood-stream. Tincture of strophanthus is a popular remedy. The active principle (strophanthin) of this drug has been widely used lately alone, that is, uncombined with anything else. It may be given internally or intravenously, the subcutaneous injection giving great pain, which, however, soon passes off, especially if the injection be made into the buttock. Personally, I have seen no special advantage in the internal administration of strophanthin as compared with tinct. strophanthi. Administered as an intravenous or intramuscular injection strophanthin acts energetically, but not in any way better than digalen. It did appear in one case of mine to act better than digalen, but only temporarily, its effects diminishing later on, whilst digalen soon reëstablished compensation. In those cases which relapse after digitalis is discontinued, and the muscular insufficiency with increased pulse-rate and arrhythmia reappear, the use of small doses of digitalis (folia digit., 0.15 to 0.25 gram *pro die;* digalen, 10 to 20 drops daily) for several months is of advantage, or the long-continued use of the same drug can do good in certain cases of muscular tachycardia and arrhythmia, where the taking of larger doses for shorter periods has had no effect. Salts of caffeine and theobromine can be taken along with digitalis and strophanthus and may be considered as vasomotor remedies. I prefer diuretin and of this 2.0 grams per day in subdivided doses. This acts as regulator of blood-pressure, and perhaps acts favorably on the vasomotor mechanism of the heart musculature itself. The tachycardia and arrhythmia in arteriosclerotic myocarditis demand the continued use of the iodides (sodium iodide—iodipin). If there be justifiable ground for suspecting syphilitic myocarditis there need be no hesitation in prescribing the necessary mercurial course. In the palpitation of those suffering from arteriosclerosis, where the heat has to combat the greatly increased arterial resistance, and particularly if accompanied with dyspepsia, Huchard recommends a milk-vegetable diet along with digitalis in small doses, and erythrol tetranitrate (in tablets 0.06 gram *pro dosi*). Small doses of morphia or codeine act favorably under certain circumstances in these cases.

Romberg draws attention to the occurrence of tachycardia in sclerosis of the coronary arteries, the treatment of which would conform to that of general arteriosclerosis. The tachycardia of aortic insufficiency merits separate discussion. Many patients afflicted with this disease show a persistently increased pulse rate as a sign of the irritable weakness of the cardiac muscle. The danger underlying this condition is the degeneration of the muscle and the insufficient emptying of the ventricle, which may give occasion to formation of a thrombus. The long-continued use of small doses of digitalis is well adapted to such cases.

In contracted kidney the action of the heart may be very much accelerated, and then it is practically always accompanied with confused rhythm (galiprhythmus). Beside a strict kidney diet, the following are of advantage: diuretin or theophylline, digitalis, camphor, caffeine small doses of codeine or morphia, hot hand and foot baths and abstraction of blood.

The occurrence of pendulum rhythm, combined with tachycardia (embryocardia), is very important and always reveals a condition of serious cardiac muscular debility and demands the energetic use of digitalis caffeine (injection of caffein-natriobenzoicum, 2 grams; aq. dist., 8 grams—a Pravaz's syringeful every two hours) and oil of camphor.

---

A NOTE ON POISONING BY EGG.—D. J. M. Miller of Atlantic City, N. J., describes several cases in which the use of even small quantities of the white or yolk of an egg has caused symptoms similar to ptomain poisoning. It makes no difference apparently how fresh the eggs are. The author believes that germs of putrefaction do penetrate the egg shells, and the poisons produced act on the very sensitive stomach of the patient. It is really ptomain poisoning that occasions the apparent idiosyncrasy.—*Medical Record,* March 13, 1909.

